# Phosphate of Lime as a Therapeutic Agent

**Published:** 1877-04

**Authors:** 


### ARTICLE XII.

### *On the Value of Phosphate of Lime as a Therapeutic Agent.*

MM. Paquelin and Jolly, of Paris, publish in the *Bulletin Generale de Therapeutique*, June 15, 1876, a paper in which they give the results of their examination into the origin of phosphate of lime in the system, and the mode by which it is eliminated by the urinary and intestinal passages and, consequently, to estimate the value which this salt may possess as a therapeutic agent. MM. Paquelin and Jolly show that the first portion of the digestive passages exercises no action on phosphate of lime, except to convert it into a superphosphate, and when this latter has reached the intestine and meets the pancreatic and intestinal fluids, it is precipitated again in the form of insoluble phosphate. The authors have made researches which prove that, although the phosphate of lime is condensed in great quantity in the bones, the other organs only contain traces of it, the bile, however, affording a large proportion. Phosphate of lime is not capable of absorption, and it has not been shown that when given to animals, whether in the soluble or insoluble state, it passes away in the stools. The authors consider that lime finds its way into the system in the form of carbonate, contained either in the solid food or in the drink, and that it forms phosphate of lime in the system by double exchange with the alkaline phosphate taken also with the food. So, as to the urinary phosphates they consider them to be formed in the urinary bladder and the biliary phosphates in the gall bladder. The conclusions they draw are the following: 1. That phosphate of lime is incapable of absorption, except in small quantity. 2. The organism consumes in general very little of this salt. 3. The circulation conveys only insignificant quantities of it, and the tissues, except the bones, contain only some traces. 4. Lime enters the organism in two states, viz., in small quantity as a superphosphate, and in rather a large proportion in non-phosphoric salts. These latter partly exist in the food as carbonate, and partly are produced by the decomposition of the alimentary phosphate of lime by the acid of digestion, such as chloride of calcium, lactate of lime, etc. 5. The system forms its phosphate of lime by double decomposition, and finds in the food all the elements necessary to increase, according to necessity, the production of this substance. The phosphate of lime in the urine is in the greater part an intra-vesical formation, and therefore the total amount of the urinary phosphate is not the direct product of dissimilation. 7. The artificial phosphates of lime, soluble or insoluble, are eliminated by the excretory passages without being utilized. 8. The addition of these phosphates to the food is an obstacle to nutrition; and, 9, the soluble preparations of phosphate of lime (superphosphates) acts as acid principles.

In the succeeding number of the *Bulletin* (June 30) the authors of the above mentioned paper modify their conclusions (7) and (9) in the following sense: 7. Of the two constituents of the phosphates of lime, namely, phosphoric acid and lime, the first is absorbed in a certain proportion in the state of alkaline phosphate, and the second is eliminated directly and almost entirely by the intestinal passages. 9. The soluble preparations of phosphate of lime act primarily as acid principles, and then by reason of the changes they undergo in the intestines, they act, secondarily in a certain degree of phosphatic agents of another base.—*British and Foreign Med.-Chir. Review.*

---

